# Effectiveness of Recruitment Strategies of Latino Smokers: Secondary Analysis of a Mobile Health Smoking Cessation Randomized Clinical Trial

**DOI:** 10.2196/34863

**Published:** 2022-06-27

**Authors:** Evelyn Arana-Chicas, Francisco Cartujano-Barrera, Katherine K Rieth, Kimber K Richter, Edward F Ellerbeck, Lisa Sanderson Cox, Kristi D Graves, Francisco J Diaz, Delwyn Catley, Ana Paula Cupertino

**Affiliations:** 1 Department of Surgery University of Rochester School of Medicine & Dentistry Rochester, NY United States; 2 Department of Public Health Sciences University of Rochester School of Medicine & Dentistry Rochester, NY United States; 3 Department of Otolaryngology University of Rochester School of Medicine & Dentistry Rochester, NY United States; 4 Department of Population Health The University of Kansas Medical Center Kansas City, KS United States; 5 Department of Oncology Georgetown University Medical Center Washington DC, DC United States; 6 Department of Biostatistics & Data Science The University of Kansas Medical Center Kansas City, KS United States; 7 Center for Children’s Healthy Lifestyle and Nutrition Children's Mercy Kansas City Kansas City, MO United States

**Keywords:** smoking cessation, Latino health, Latino recruitment, health disparities, participant recruitment

## Abstract

**Background:**

Latinos remain disproportionately underrepresented in clinical trials, comprising only 2%-3% of research participants. In order to address health disparities, it is critically important to increase enrollment of Latino smokers in smoking cessation trials. There is limited research examining effective recruitment strategies for this population.

**Objective:**

The purpose of this study was to compare the effectiveness of direct versus mass and high- versus low-effort recruitment strategies on recruitment and retention of Latino smokers to a randomized smoking cessation trial. We also examine how the type of recruitment might have influenced the characteristics of enrolled participants.

**Methods:**

Latino smokers were enrolled into *Decídetexto* from 4 states—New Jersey, Kansas, Missouri, and New York. Participants were recruited from August 2018 until March 2021. Mass recruitment strategies included English and Spanish advertisements to the Latino community via flyers, Facebook ads, newspapers, television, radio, church bulletins, and our *Decídetexto* website. Direct, high-effort strategies included referrals from clinics or community-based organizations with whom we partnered, in-person community outreach, and patient registry calls. Direct, low-effort strategies included texting or emailing pre-existing lists of patients who smoked. A team of trained bilingual (English and Spanish) recruiters from 9 different Spanish-speaking countries of origin conducted recruitment, assessed eligibility, and enrolled participants into the trial.

**Results:**

Of 1112 individuals who were screened, 895 (80.5%) met eligibility criteria, and 457 (457/895, 51.1%) enrolled in the trial. Within the pool of screened individuals, those recruited by low-effort recruitment strategies (both mass and direct) were significantly more likely to be eligible (odds ratio [OR] 1.67, 95% CI 1.01-2.76 and OR 1.70, 95% CI 0.98-2.96, respectively) and enrolled in the trial (OR 2.60, 95% CI 1.81-3.73 and OR 3.02, 95% CI 2.03-4.51, respectively) compared with those enrolled by direct, high-effort strategies. Among participants enrolled, the retention rates at 3 months and 6 months among participants recruited via low-effort strategies (both mass and direct) were similar to participants recruited via direct, high-effort methods. Compared with enrolled participants recruited via direct (high- and low-effort) strategies, participants recruited via mass strategies were less likely to have health insurance (44.0% vs 71.2% and 71.7%, respectively; *P*<.001), lived fewer years in the United States (22.4 years vs 32.4 years and 30.3 years, respectively; *P*<.001), more likely to be 1st generation (92.7% vs 76.5% and 77.5%, respectively; *P*=.007), more likely to primarily speak Spanish (89.3% vs 65.8% and 66.3%, respectively), and more likely to be at high risk for alcohol abuse (5.8 mean score vs 3.8 mean score and 3.9 mean score, respectively; *P*<.001).

**Conclusions:**

Although most participants were recruited via direct, high-effort strategies, direct low-effort recruitment strategies yielded a screening pool more likely to be eligible for the trial. Mass recruitment strategies were associated with fewer acculturated enrollees with lower access to health services—groups who might benefit a great deal from the intervention.

**Trial Registration:**

ClinicalTrials.gov identifier: NCT03586596; https://clinicaltrials.gov/ct2/show/NCT03586596

**International Registered Report Identifier (IRRID):**

RR2-DOI: 10.1016/j.cct.2020.106188

## Introduction

Latinos constitute the largest minority group in the United States, representing 18.5%, or 55 million, of the current US population [1], and this group is projected to grow to 30% by 2060 [2]. An estimated 6 million Latinos (9.8%) in the United States are current cigarette smokers [3,4]. Although the National Institutes of Health (NIH) Revitalization Act of 1993 [5,6] called for the inclusion of minorities in clinical research, Latinos remain disproportionately underrepresented in clinical trials, comprising only 2%-3% of research participants [7-9].

Increasing enrollment of Latino smokers in smoking cessation trials is critical for addressing health disparities; however, there is limited research examining effective strategies for recruiting this population [10,11]. Common obstacles to recruitment may include language barriers and health literacy [12,13], and some Latinos may have concerns or mistrust of government-funded research related to privacy or deportation concerns [7,13-15]. Increased burden from social conditions such as poverty [16], low education levels [16,17], and immigration issues [18] also contribute to low participation in clinical trials. These reported barriers may lead to the perception that recruitment of Latinos into clinical trials is difficult. However, despite these barriers, when invited to participate in research, enrollment rates of Latinos are comparable to those of non-Latino Whites [7,14]. Indeed, Latinos are interested in enrolling in research when recruitment strategies are culturally and linguistically tailored to them.

Literature on the recruitment of Latinos into clinical trials has described the use of different recruitment strategies [19-23]. Some studies have recruited Latinos through proactive recruitment in which study staff directly contact individual potential participants [20,24] and reactive recruitment in which studies disseminate information via mass media and potential participants must contact the study themselves [19,21,25]. Often, recruitment studies emphasize including Latino researchers, fostering community connections to build trust, and using culturally and linguistically tailored recruitment materials delivered through culturally appropriate outlets such as Latino newspapers [19,22,23].

Traditional categorizations of recruitment approaches (eg, into proactive versus reactive) do not capture the complexity of current recruitment strategies. Although proactive recruitment strategies involving personal outreach to individuals have historically necessitated relatively high effort compared with reactive outreach efforts such as mass advertising, the advent of electronic communications such as text messages and emails now allows direct, personalized outreach with relatively low effort. To date, there has been a lack of research distinguishing the effects of direct versus mass outreach and level of effort on recruitment success. Furthermore, there are limited data available on the retention of Latinos who were recruited via different strategies in clinical trials. One study compared ethnic-specific retention rates in various clinical trials and found that Latino adults have a retention rate of ~54% in clinical trials, and this did not significantly differ compared with other ethnic groups [26]. Only one study has analyzed the effects of recruitment type on retention; however, the recruitment strategies used in that study were limited to newspapers, posters on buses and subways, study flyers at community organizations, and in-person recruitment and community organizations [24].

It is also possible that different recruitment strategies will yield participants with different characteristics. For example, compared with direct recruitment, mass media recruitment (eg, radio, flyers) may yield more inherently motivated participants since little outreach or encouragement is provided; those who reactively join the study following mass media exposure may have higher commitment to behavior change [27]. This study, therefore, calculated the associations of mass versus direct recruitment strategies, involving high and low study staff effort, with characteristics of Latino smokers who were screened, enrolled, and retained in a randomized smoking cessation trial—*Decídetexto* [28].

## Methods

### Study Design

This study is a secondary data analysis of *Decidetexto*, a mobile health (mHealth) smoking cessation randomized clinical trial. It compares the efficiency ratios for eligibility, enrollment, and retention (at 3 months and 6 months) of Latino smokers recruited via direct versus mass and high- versus low-effort recruitment strategies.

### Ethical Approval

The details of the study intervention and protocol are described elsewhere [28]. Study procedures were approved and monitored by Hackensack University Medical Center (#Pro2017-0528), the University of Rochester Medical Center (IRB #STUDY00005080), and the University of Kansas Medical Center Institutional Review Boards (IRB # KUMC IRB #STUDY00004475).

### Recruitment

Latino smokers were enrolled into *Decídetexto* from multiple communities (both urban and rural) in 4 states—New Jersey, Kansas, Missouri, and New York. Participants were recruited from August 2018 until March 2021. Direct recruitment strategies involved one-on-one communication with identified Latino smokers and were dichotomized as either “direct, high-effort” or “direct, low-effort” strategies. Recruitment and eligibility were conducted by a team of trained bilingual (English and Spanish) recruiters from different countries of origin (eg, Cuba, Dominican Republic, Ecuador, El Salvador, Mexico, Nicaragua, Peru, Puerto Rico, Venezuela).

In this study, “recruitment method” refers broadly to either mass, direct high-effort, or direct low-effort recruitment methods. “Recruitment strategies” refer to the specific recruitment strategy implemented. Mass recruitment strategies did not rely on interpersonal communication but instead included bilingual (English and Spanish) advertisements of the study to the larger Latino community via flyers, Facebook ads, newspapers, television, radio, church bulletins, and the *Decídetexto* website. Direct, high-effort strategies required more staff resources to connect with potential participants and included personal calls based on referrals from clinics or community-based organizations (CBOs), in-person community outreach, and personal calls made to patients on patient registries. Furthermore, as reported in previous research [23], research staff adhered to important cultural values in their interactions with potential participants by communicating with *personalismo* (initiating warm conversations that conveyed care and understanding of the patient’s circumstances), *simpatía* (not criticizing the patient), and *confianza* (establishing trust). Direct, low-effort strategies demanded less time and effort from the research team. Direct, low-effort strategies included sending emails and texts to patients on patient registries and referrals from family and friends. Direct, low-effort and mass strategies were similar in that interested participants had to take the step of contacting the study for screening and follow-up. In this sense, they are both “reactive” recruitment strategies. However, in this study, they are differentiated by whether the recruitment strategy used mass communication to the Latino community or was directly sent to an identified Latino smoker.

### Measures

Research staff administered all study assessments either in person or via telephone. Prior to completing the eligibility questionnaire, participants were asked the open-ended question “How did you learn about the study?” The baseline assessment collected data on demographics (eg, gender, education, age, income, health insurance status, marital status), smoking characteristics (eg, cigarettes smoked per day; the number of past quit attempts), and biopsychosocial variables: eg, depressive symptoms via the Patient Health Questionnaire-2 (PHQ-2) scale [29], alcohol use via the Alcohol Use Disorders Identification Test-2 (AUDIT-2) [30], anxiety via the Generalized Anxiety Disorder-2 (GAD-2) [31], self-efficacy [32], and acculturation measures including years lived in the United States, primary language, generation, and region of origin.

### Analyses

Logistic regression analyses were used to calculate odds ratios (ORs; efficiency ratios) and 95% CIs for associations (1) between recruitment method and obtaining eligible individuals among screened individuals, (2) between recruitment method and enrolling the screened participants, and (3) between recruitment method and retaining the enrolled participants at the 3-month and 6-month follow-up visits. Rates of eligibility, enrollment, and retention across the 3 recruitment methods and recruitment strategies were compared using chi-square tests. For each recruitment method, characteristics of enrolled participants were summarized with percentages for categorical variables and with means and SDs for continuous variables. Differences in categorical variables were exploratorily compared using Pearson chi-square tests while differences in continuous variables were compared using 1-way ANOVA tests. Reasons for ineligibility were compared between participants who were recruited via direct, high-effort; direct, low-effort; and mass recruitment methods using Pearson chi-square tests or Fisher exact tests. Data were analyzed using SPSS version 25.

## Results

### Overview

Of 1112 individuals who completed screening, 895 (80.5%) met eligibility criteria, and 457 (457/895, 51.1%) enrolled in the trial. The majority of participants were enrolled via direct, high-effort strategies (300/457, 65.6%). Table 1 lists the numbers screened, eligible, enrolled, and retained at 3 months and 6 months by recruitment method and includes efficiency ratios for eligibility, enrollment, and retention at 3 months and 6 months.

Table 2 shows the efficiency of specific recruitment strategies. Overall, eligibility efficiency ratios were lowest for Facebook ads (66.7%), followed by in-person community outreach (74.7%), our Decidetexto website (75.0%), and patient registry calls (79.1%). Enrollment efficiency ratios were lowest for Facebook ads (33.3%), followed by television (41.9%), in-person community outreach (31.9%), and patient registry calls (35.4%). The 3-month retention efficiency ratios were lowest for the Decidetexto website (66.7%), television (69.2%), and patient registry text (70.6%). The 6-month retention efficiency ratios were lowest for television (76.9%) and patient registry text (76.5%).

Compared with the direct, high-effort recruitment method, individuals screened in both the mass and direct, low-effort recruitment methods were significantly more likely to be eligible (OR 1.67, 95% CI 1.01-2.76 and OR 1.70, 95% CI 0.98-2.96, respectively) and enrolled (OR 2.60, 95% CI 1.81-3.73 and OR 3.02, 95% CI 2.03-4.51, respectively; Table 3). Of participants enrolled, those recruited via mass and direct, low-effort methods were just as likely to be retained at 3 months and 6 months compared with participants recruited via the direct, high-effort method. Furthermore, given that 45.5% (208/457) of all enrolled participants were recruited via patient registry calls and that 69.3% (208/300) of all participants who were recruited via direct, high-effort strategies were recruited via patient registry calls, a logistic regression model was run to identify any differences in efficiency ratios between patient registry calls and other direct, high-effort strategies. No differences were found between the 2 (data not shown).

**Table 1:** Efficiency ratios for personalized and nonpersonalized recruitment methods.

Recruitment method	Number screened^a^	Number eligible^b^	Number enrolled	Number retained at 3 months	Number retained at 6 months	Eligibility efficiency ratio^c^, %	Enrollment efficiency ratio^d^, %	3-month retention efficiency ratio^e^, %	6-month retention efficiency ratio^f^, %
Mass	144	127	84	69	73	88.2	58.3	82.1	86.9
Direct, low effort	117	101	73	60	61	86.3	62.4	82.2	83.6
Direct, high effort	847	666	300	261	261	78.6	35.4	87.0	87.0

^a^The total is not 1112 because of missing data on the recruitment strategy.

^b^The total is not 895 because of missing data on the recruitment strategy.

^c^Ratio of number eligible to number screened.

^d^Ratio of number enrolled to number screened.

^e^Ratio of number retained at 3 months to number enrolled.

^f^Ratio of number retained at 6 months to number enrolled.

**Table 2:** Recruitment efficiency of specific recruitment strategies.

Recruitment method			Proportion for the recruitment strategies, n (%)		Number screened		Number eligible		Number enrolled		Number retained at 3 months		Number retained at 6 months		Eligibility efficiency ratio, %		Enrollment efficiency ratio, %		3-month retention efficiency ratio, %		6-month retention efficiency ratio, %
**Mass (n=144)**																					
	Church bulletin	4 (2.8)		4		4		4		4		3		100		100		100		75.0	
	Newspaper	22 (15.3)		22		22		16		14		14		100		72.7		87.5		87.5	
	Radio	32 (22.2)		32		28		18		14		15		87.5		56.3		77.8		83.3	
	Flyer	48 (33.3)		48		40		29		25		27		83.3		60.4		86.2		93.1	
	Decídetexto website	4 (2.8)		4		3		3		2		3		75.0		75.0		66.7		100	
	Television	31 (21.5)		31		24		13		9		10		77.4		41.9		69.2		76.9	
	Facebook ads	3 (2.1)		3		2		1		1		1		66.7		33.3		100		100	
**Direct, low effort (n=117)**																					
	Clinic or CBO^a^ email	10 (8.5)		10		9		7		7		6		90		100		100		85.7	
	Patient registry text	35 (29.9)		35		28		17		12		13		80.0		48.6		70.6		76.5	
	Friend or family referral	72 (61.6)		72		64		49		41		42		88.9		68.1		83.7		85.7	
**Direct, high effort (n=847)**																					
	Clinic or CBO referral	81 (9.6)		81		68		35		30		29		83.9		43.2		85.7		82.3	
	In-person community outreach	182 (21.5)		182		136		58		48		52		74.7		31.9		82.3		89.7	
	Patient registry call	584 (68.9)		584		462		207		183		180		79.1		35.4		88.4		86.9	

^a^CBO: community-based organization.

**Table 3:** Results of the logistic regression analysis using recruitment method to predict eligibility, enrollment, and retention.

Recruitment method	Eligible^a^ (n=895)			Enrolled^a^ (n=459)			Retained at 3 months^b^ (n=390)			Retained at 6 months^b^ (n=395)	
	Odds ratio (95% CI)	*P* value	Odds ratio (95% CI)		*P* value	Odds ratio (95% CI)		*P* value	Odds ratio (95% CI)		*P* value
Mass recruitment	1.67 (1.01-2.76)	.04	2.60 (1.81-3.73)		<.001	1.4 (0.76-2.80)		.26	1.01 (0.49-2.1)		.98
Direct, low effort	1.70 (0.98-2.96)	.06	3.02 (2.03-4.51)		<.001	1.4 (0.73-2.9)		.29	1.32 (0.65-2.66)		.44
Direct, high effort	1.0	N/A^c^	1.0		N/A	1.0		N/A	1.0		N/A

^a^Denominator for recruitment method is number screened.

^b^Denominator for recruitment method is number enrolled.

^c^N/A: not applicable.

### Differences in Participant Characteristics

The characteristics of enrolled participants (Table 4) were compared across recruitment methods. Participants recruited via mass recruitment strategies were significantly less likely to have health insurance (44.0% vs 71.2% and 71.7%, respectively; *P*<.001), lived significantly fewer years in the United States (22.4 years vs 32.4 years and 30.3 years, respectively; *P*<.001), significantly more likely to be 1st generation (92.7% vs 76.5% and 77.5%, respectively; *P*=.007), significantly more likely to primarily speak Spanish (89.3% vs 65.8% and 66.3%, respectively), and significantly more likely to be at high risk for alcohol abuse (5.8 mean score vs 3.8 mean score and 3.9 mean score, respectively; *P*<.001) compared with those recruited via direct, low-effort and direct, high-effort strategies. Participants recruited via mass recruitment strategies were significantly more likely to come from Mexico (45.2% vs 20.5% and 8.3%, respectively; *P*<.001), while participants from Central America were more likely to be recruited via direct, low-effort strategies and direct, high-effort strategies (13.1% vs 32.9% and 35.0%, respectively; *P*<.001) compared with mass recruitment strategies. Participants born in the United States were significantly more likely to be recruited via both direct low-effort strategies and direct high-effort strategies (9.5% vs 28.8% and 25.8%, respectively; *P*<.001) compared with mass recruitment strategies. Moreover, Latino smokers recruited via direct, high-effort strategies were more likely to have depressive symptoms (1.7 mean score vs 1.1 mean score and 1.4 mean score, respectively; *P*=.02) and anxiety (1.8 mean score vs 1.1 mean score and 1.6 mean score, respectively; *P*=.004).

With respect to the 3-month retention rate, participants were significantly more likely to primarily speak English (68.7% vs 80.6%; *P*=.06) and to be a second or higher generation American (75.8% vs 89.6%; *P*=.02) compared with participants who did not complete their 3-month follow-up assessment. With respect to 6-month retention, particpants were significantly older (49.7 years vs 46.5 years; *P*=.02) and reported less self-efficacy (1.9 mean score vs 2.2 mean score; *P*=.008) compared with participants who did not complete their 6-month follow-up assessment (Table 4).

Of participants who were ineligible (n=217), the most frequent reasons for ineligibility were planning to move in the next 6 months, not willing to come to all study visits, smoking on average less than 3 cigarettes per day, and not knowing how to send or read text messages (Table 5). Ineligible participants identified via direct, high-effort strategies were significantly more likely to plan to move in the next 6 months (69.4% vs 13.9% and 16.7%, respectively; *P*=.04) compared with mass and direct, low-effort strategies.

**Table 4:** Baseline characteristics of enrolled participants who were recruited using proactive and reactive strategies and who were retained at 3 months and 6 months.

Characteristic		Recruitment method					Retained at 3 months (n=391)						Retained at 6 months (n=395)				
		Mass (n=144), n (%)	Direct, low effort (n=117), n (%)	Direct, high effort (n=847), n (%)	*P* value	Yes, n (%)		No, n (%)	*P* value		Yes, n (%)			No, n (%)		*P* value	
Female		54 (64.3)	39 (53.4)	157 (52.3)	.15	211 (54.1)		39 (58.2)	.60		212 (53.7)			38 (61.3)		.28	
Greater than a high school education		25 (29.8)	29 (39.7)	117 (39.0)	.28	152 (39.0)		19 (28.4)	.10		153 (38.7)			18 (29.0)		.16	
Has health insurance		37 (44.0)	52 (71.2)	215 (71.7)	<.001	264 (68.2)		40 (59.7)	.21		266 (67.9)			38 (61.3)		.31	
Married		52 (61.9)	35 (47.9)	157 (52.3)	.22	207 (53.4)		37 (56.1)	.69		206 (52.4)			38 (62.3)		.17	
Employed full time		55 (65.5)	36 (49.3)	151 (50.3)	.04	212 (54.4)		30 (44.8)	.19		209 (52.9)			33 (53.2)		.99	
**Annual income (US $)**																	
	0-29,000	33 (39.3)	29 (39.7)	125 (41.7)	.34	159 (42.0)		29 (46.8)	.53		165 (43.8)			22 (36.1)		.45	
	30,000-59,000	33 (39.3)	22 (30.1)	86 (28.7)		125 (33.2)		16 (25.8)			120 (31.8)			21 (34.4)			
	≥60,000	13 (15.5)	20 (27.4)	77 (25.7)		93 (24.7)		17 (27.4)			92 (24.4)			18 (29.5)			
Age (years)^a^		46.6 (11.1)	50.1 (12.5)	48.9 (10.7)	.11	48.9 (11.1)		47.5 (11.2)	.35		49.7 (11.1)			45.6 (10.9)		.02	
Number of cigarettes per day^a^		10.3 (7.6)	12.8 (9.3)	11.7 (7.8)	.17	11.5 (7.9)		12.0 (8.0)	.68		11.5 (7.9)			12.4 (8.5)		.37	
Number of prior quit attempts^a^		5.0 (9.4)	2.7 (5.6)	3.5 (7.8)	.16	3.8 (8.2)		2.7 (4.8)	.30		3.9 (8.3)			2.4 (3.4)		.17	
Alcohol score^b^		5.8 (2.6)	3.8 (2.4)	3.9 (2.5)	<.001	4.2 (2.6)		4.5 (2.7)	.44		4.2 (2.6)			4.5 (2.8)		.38	
Depressive symptoms^b^		1.1 (1.6)	1.4 (1.5)	1.7 (1.8)	.02	1.5 (1.7)		1.4 (1.8)	.65		1.6 (1.7)			1.3 (1.6)		.29	
Anxiety^b^		1.1 (1.4)	1.6 (1.7)	1.8 (1.7)	.004	1.7 (1.7)		1.6 (1.7)	.60		1.7 (1.7)			1.6 (1.6)		.60	
Self-efficacy^a^		2.1 (0.82)	2.0 (0.87)	1.9 (0.71)	.13	1.9 (0.7)		2.0 (1.7)	.72		1.9 (0.7)			2.2 (0.9)		.008	
Years in the United States^a^		22.4 (14.3)	32.4 (16.9)	30.3 (16.7)	<.001	29.4 (17.0)		27.8 (13.7)	.43		29.2 (16.9)			29.1 (14.7)		.96	
Language, Spanish		77 (89.3)	48 (65.8)	199 (66.3)	<.001	268 (68.7)		54 (80.6)	.06		275 (69.6)			47 (75.8)		.37	
1st generation		76 (92.7)	52 (76.5)	224 (77.5)	.007	292 (75.8)		60 (89.6)	.02		305 (78.0)			47 (77.0)		.87	
**Region of birth**																	
	Mexico	38 (45.2)	15 (20.5)	25 (8.3)	<.001	64 (16.5)		14 (20.9)	.07		67 (17.0)			11 (17.7)		.04	
	Caribbean	11 (13.1)	24 (32.9)	105 (35.0)		120 (30.9)		20 (29.9)			126 (31.9)			14 (22.6)			
	South America	18 (21.4)	11 (15.1)	84 (28.0)		91 (23.5)		22 (32.8)			98 (24.8)			15 (24.2)			
	Central America	9 (10.7)	2 (2.7)	8 (2.7)		15 (3.9)		4 (6.0)			12 (3.0)			7 (11.3)			
	United States	8 (9.5)	21 (28.8)	76 (25.8)		98 (25.3)		7 (10.4)			90 (22.8)			15 (24.2)			

^a^Mean (SD).

^b^Score (sum score).

**Table 5:** Major reasons for ineligibility by recruitment method (only ineligibility criteria that included a total of ≥10 individuals are reported).

Reasons	Mass^a^ (n=20), n (%)	Direct, low effort^a^ (n=16), n (%)	Direct, high effort^a^ (n=180), n (%)	*P* value
Not willing to come to all study visits	6 (30.0)	1 (6.7)^b^	41 (25.6)^c^	.22
Does not know how to send or read text messages	4 (20.0)	1 (6.7)^b^	36 (21.4)^d^	.51
Smokes cigarettes less than 3 days/week	4 (20.0)	3 (18.8)	36 (20.2)^e^	.20
Planning to move in the next 6 months	5 (25.0)	6 (37.5)	25 (14.7)^f^	.04
Uses other tobacco products more than 1 day/week	5 (25.0)	4 (25.0)	24 (13.9)^g^	.23
Not interested in quitting in 30 days	2 (10.0)	1 (6.7)	9 (5.3)^h^	.41
Has not smoked cigarettes for at least 6 months	2 (10.0)	1 (6.7)	7 (4.0)^i^	.29

^a^The denominator is the difference across ineligibility criteria because of missing data.

^b^n=15.

^c^n=160.

^d^n=168.

^e^n=178.

^f^n=170.

^g^n=172.

^h^n=82.6.

^i^n=174.

## Discussion

### Principal Findings

This paper compared mass; direct, low-effort; and direct, high-effort recruitment methods on Latino eligibility, enrollment, and retention at 3 months and 6 months for a smoking cessation clinical trial, *Decídetexto*. Results showed that, although direct, high-effort methods yielded the highest total number of enrollees, eligibility and enrollment were significantly lower when compared with the mass and direct, low-effort methods. However, when considering retention at 3 months and 6 months, there is no evidence that method of recruitment impacted retention once participants were enrolled in the study. Thus, although the eligibility and enrollment rates were low for direct, high-effort strategies, participants are just as likely to be retained after they are enrolled when compared with mass and direct low-effort strategies.

It is important to note that, although mass and direct, low-effort strategies are efficient methods and do not demand much staff time, they are unlikely to reach the recruitment goal for a randomized clinical trial without contribution from direct, high-effort strategies. Future studies should include cost-effectiveness to determine whether highly funded mass and direct, low-effort strategies can recruit equal numbers in a cost-effective manner. This is especially important to consider as mass recruitment strategies seemed to yield fewer acculturated enrollees with lower access to health services—groups that might benefit a great deal from the intervention.

Our research corroborates a study that tested the efficiency of strategies to recruit Latino male smokers. That study found that reactive recruitment was more efficient than proactive recruitment but yielded significantly fewer participants and was costlier per participant enrolled [11]. As noted by Harris et al [27], reactive recruitment may be more effective at identifying eligible individuals because it reaches a wider audience and individuals who take the trouble to respond are likely to be more ready and motivated to quit. Furthermore, individuals who learn about research via mass media may have more time to collect information about the study and consider the pros and cons of enrolling before calling the study phone number to complete eligibility. This, in turn, might prevent less motivated individuals from contacting the study for screening. It should be noted that this study’s advertisements did not include all of our eligibility criteria. Potential participants were able to self-screen for some criteria using the Latino identity and current smoker criteria that were noted in the advertisments. There were a number of additional criteria they had to meet (Table 5). Future research should consider enhancing advertisements (eg, flyers, posters) to yield higher response rates using theoretical constructs such as self-efficacy, social norms, and rewards. Moreover, additional research should consider assessing which method of recruitment yielded a higher rate of participants who quit smoking.

Of the individual mass recruitment strategies, Facebook ads yielded the lowest efficiency ratios for eligibility and enrollment. This contradicts previous research that found Facebook was a useful recruitment tool for smokers [33,34]. Of direct low-effort strategies, patient registry texts yielded the lowest efficiency ratios for eligibility and enrollment and yielded among the lowest ratios for retention at 3 months and 6 months. This is consistent with previous research reporting an ~34% enrollment rate for patients recruited via text messages [35]. It is interesting to note that referring friends and family members were either (1) Latino smokers on a patient registry who received a call from us but no longer smoked or were ineligible or (2) study participants who had completed the study. Of direct, high-effort strategies, in-person community outreach yielded the lowest efficiency ratios for eligibility and enrollment. This is important to note, as this is the recruitment strategy that demanded the most staff resources. Although patient registry calls yielded the highest number of participants, it was among the lowest efficiency ratio for eligibility and enrollment. This is in contrast to previous research that has reported the feasibility and cost-effectiveness of recruiting participants via calls from patient registries via a research associate program [20,33].

With respect to the characteristics of participants recruited via the different recruitment methods, mass recruitment yielded less acculturated participants (eg, more likely to speak Spanish, to be 1st generation, fewer years lived in the United States) who were more likely to be at risk for alcohol abuse than participants recruited via direct, low-risk and direct, high-risk methods. It is possible that low-acculturation Latino smokers face unique barriers that limit the effectiveness of direct recruitment strategies.

We also found that Latinos from different Latin American regions appear to respond differently to different recruitment methods. Mexicans were more likely to be recruited via mass recruitment strategies compared with all other Latin American regions, while Latinos from the Caribbean were more likely to be recruited using direct strategies. Thus, recruitment approaches that researchers choose to employ should be determined by their population of interest and the desired participant characteristics. Furthermore, Latino smokers experiencing depression or anxiety were less likely to respond to mass recruitment. This may be because they are less motivated to quit smoking or they have less energy to reach out to inquire about the study [36]. The psychosocial finding corroborates findings from a study that compared reactive versus proactive recruitment strategies in recruiting African American smokers [27], in which it was found that participants recruited proactively were more likely to report indicators of depression. Taken together, these findings suggest that both mass and direct recruitment strategies should be implemented for studies interested in recruiting Latino participants across the socioeconomic, acculturation, and country of origin spectra. Additional research is also needed to examine differences in clinical outcomes based on recruitment method.

### Limitations

The *Decídetexto* clinical trial was not designed to test the efficiency of recruitment strategies; therefore, this study has several limitations. Given the broad reach of our advertisements, it is possible that participants were exposed to multiple advertisement strategies. It is possible that individuals responding to a mass strategy may have been exposed to a direct strategy. Therefore, some cross-contamination effect is likely to have occurred. However, no participants in this study mentioned that they learned about the study through more than one strategy. Moreover, we were unable to conduct a cost analysis in this study given that (1) most of our mass recruitment strategies were free of cost or paid in an unusual way (eg, paid a graphic artist in Mexico) and (2) we had volunteers aid in personalized recruitment for this study. We did not collect data on barriers to participant retention. Despite these limitations, this study has high representation of a heterogeneous group of Latino smokers representing different Latin American regions of origin, making it generalizable to Latinos nationwide. The study team consisted of Latino researchers and interns from different Latin American countries of origin, several of whom were native Spanish speakers. Our recruitment efforts involved working collaboratively with a community advisory board, collaborating closely with local CBOs, and culturally and linguistically tailoring all materials to Latino smokers. Furthermore, the bulk of our recruitment occurred prior to the onset of the COVID-19 pandemic. No recruitment activities occurred from March 2020 through July 2020. From August 2020 to March 2021 we recruited 21 additional participants, 62% of whom were recruited via mass recruitment strategies. It is possible that the effect of recruitment strategies will be different during and after the COVID-19 pandemic. Moreover, although this study is specific to a tobacco treatment trial, the findings are relevant to health research and clinical trials broadly.

### Conclusion

This study compared the eligibility, enrollment, and retention efficiency ratios of recruiting Latino smokers via mass; direct, low-effort; and direct, high-effort strategies utilized in *Decídetexto*, a mobile smoking cessation randomized clinical trial. A heterogeneous sample of Latino smokers was enrolled in the trial. Results show that, although direct, high-effort recruitment strategies yielded the highest total number of enrollees, eligibility and enrollment were significantly lower when compared with mass and direct, low-effort recruitment strategies. Yet, when considering retention at 3 months and 6 months, there is no evidence that method of recruitment impacted retention once participants were enrolled in the study. Participants recruited via mass recruitment strategies were less acculturated, of lower socioeconomic status, and more likely to be Mexican than those recruited via other strategies. These findings suggest that these 3 recruitment methods should be implemented for studies interested in recruiting Latino participants across the socioeconomic, acculturation, and country of origin spectra. These findings provide further insight into effective recruitment strategies for Latino smokers.

## Abbreviations

AUDIT-2: Alcohol Use Disorders Identification Test-2
CBO: community-based organization
GAD-2: Generalized Anxiety Disorder-2
mHealth: mobile health
NCI: National Cancer Institute
PHQ-2: Patient Health Questionnaire-2
